# A Pilot Study on the Effect of Thyme Microemulsion Compared with Antibiotic as Treatment of *Salmonella* Enteritidis in Broiler

**DOI:** 10.1155/2022/3647523

**Published:** 2022-02-24

**Authors:** Engy Ahmed Hamed, May Fathy Abdelaty, Hend Karam Sorour, Dalia M. A. Elmasry, Marwa Ali Abdelmagid, Mohammed Ahmed Maher Saleh, Mona Aly Abdelhalim AbdelRahman

**Affiliations:** ^1^Reference Laboratory for Veterinary Quality Control on Poultry Production, Animal Health Research Institute, Agricultural Research Center (ARC), Nadi El-Seid Street, Dokki P.O. Box 246, Giza 12618, Egypt; ^2^Nanomaterials Research and Synthesis Unit, Animal Health Research Institute, ARC, Dokki, Giza, Egypt

## Abstract

Multidrug resistance poses a global threat to the poultry industry and public health, so the direction towards eliminating the use of antibiotics and finding alternatives is a vital step to solve this problem. Thyme microemulsion (10% oil/water) had nanodrop size 28.65 ± 0.89 nm, with a polydispersity index (PDI) of 0.28 with greater homogeneity. It showed IC_50_ > 100 ug/ml on cytotoxicity assay and 14 active components by GC-Mass. The study was carried out using 210 Cobb chicks divided into fourteen groups. The infected groups were challenged using two *Salmonella* Enteritidis multidrug resistance (MDR) and *Salmonella* Enteritidis sensitive strains to the sulpha-trimethoprim antibiotic. The challenged inoculum was 1 × 10^9^ CFU of *Salmonella* Enteritidis by oral route. The MIC treatments doses were 1 ml/liter water for thyme oil and thyme microemulsion and 33.34 mg/kg b.wt sulfadiazine for 5 days. The results showed that both thymol oil (0.1%) and microemulsion (0.01%) are able to decrease the count of *Salmonella* Enteritidis in cecal content and fecal dropping and the mortality rates after five days of treatment. In addition, thyme oil and microemulsion had no pathological alteration on chickens' tissues that were collected two weeks after giving the treatment. By the robust HPLC method, the SDZ and TMP residues in tissues of infected groups treated with Cotrimazine® + thyme oil microemulsion had a slight significant economic impact (*P* < 0.05) compared to Cotrimazine® alone. In conclusion, thymol oil and microemulsion could be an alternative economic choice for multidrug resistance *Salmonella* Enteritidis treatment in poultry farms.

## 1. Introduction

Poultry is an important source of animal protein for human. *Salmonella* is considered one of the most important zoonotic bacteria which causes food-borne diseases to human, through the consumption of poultry and poultry byproducts [1–3]. On the other hand *Salmonella* causes high economic losses in poultry production due to a decrease in productivity, low egg production, and premature deaths, in addition to the high cost of *Salmonella* infection treatment. So control of *Salmonella* infection in poultry is considered very important goal for decreasing human infection and economic losses [4, 5].

*Salmonella* Enteritidis (*S.* Enteritidis) serotype is one of the most important members of 2500 *Salmonella* serovars which can be transmitted from poultry and poultry byproducts to human causing food poisoning diseases [6]; furthermore, *S.* Enteritidis became highly resistant, so prevention and control of *S.* Enteritidis infection of poultry is very important for human health and the poultry industry [7, 8].

The critical task of poultry producers is to stop the uncontrolled usage of some antibiotics as growth promoters in poultry feed [9, 10]. Unfortunately the misuse of antibiotics leads to the development of multidrug resistant bacterial strains especially in *Salmonella* species [11, 12].

Development of products rather than antibiotics is done to treat or at least stop the growth of bacteria such as essential oils (EOs) and organic acids (EOA) which have antimicrobial and antioxidant effects, improve animal intestinal health, and promote the absorption of nutrients. Nowadays these products used as antibiotic substitutes [13, 14].

The action of EOs depends on their chemical composition; generally EOs can inhibit bacterial growth, alter the permeability of cell membrane, and render the synthesis of some proteins and ATP [15]. Adding of EOs in poultry feed increases feed Conversion Ratio and improve growth performance in poultry [16].

Thymol oil is one of the major components of thyme. It represents about 47–59% of thyme and has a great effect on *Salmonella* Typhimurium [17]. Thymol oil has an effect on the synthesis of ATP leading to increase in the concentration of ATP inside the bacterial cell and lead to destruction and death of the bacterial cell, also can affect the production of protein which is involved in energy metabolism, and also changes other proteins which adversely affect survival of bacteria in adverse conditions, so the permeability of cell membrane of *Salmonella* Typhimurium is affected by thymol oil. Thymol-benzoic acid has a bacteriostatic effect on *Salmonella Enteritidis* [15, 18, 19].

The combination of the sulfadiazine (SDZ) and trimethoprim (TMP) formulation at a 5 : 1 ratio is widely used in poultry industry against different microorganisms [20]. This may lead to residues in poultry tissues with possible hazards like development of antimicrobial resistance and toxic and allergic effect in addition to diminished effect on gut microflora [21].

Microemulsions are colloidal dispersions made up of small oil droplets (20–200 nm) suspended in water. The microemulsions must be properly constructed to ensure that they remain physically stable during storage and use [22]. Antimicrobial microemulsions are emulsified oil, water, and detergent combinations that have been demonstrated to have wide activity against enveloped viruses, bacteria, and fungus [23], at harmless quantities in animals. Microemulsions' antibacterial activity is unlike antibiotics', nonspecific, allowing for broad-range action while decreasing the potential for resistance progress. When microemulsions bind to lipid bilayers in cell membranes, the energy contained in the oil-and-detergent emulsion is released, destabilizing the bacteria's lipid membrane; therefore, their antibacterial action developed [24].

Infection of chicken with *Salmonella* Typhimurium leads to severe pathological changes in cecal wall like sever inflammation and degeneration in cecal wall treatment with thymol oil and thymol microemulsion leading to high improvement of histopathological picture of cecal wall of infected chickens [25].

Aim of this study is trying to use thymol oil and thymol microemulsion for controlling of *Salmonella* Enteritidis infection with/without the sulpha-trimethoprim antibiotics in broilers and show its residual and histopathological effect in broiler tissues in comparison with the sulpha-trimethoprim antibiotics.

## 2. Materials and Methods

### 2.1. Treatments

#### 2.1.1. Thymol Oil (100%)

It was purchased from Alamal for natural oil, Gharbia governorate, Egypt.

#### 2.1.2. Thymol Microemulsion (10%)

*(1) Preparation of Thymol Microemulsion*. Thymol oil was purchased from Alamal for natural oil, Gharbia, Tween 80, deionized water, propylene glycol, and sodium chloride were obtained from Sigma-Aldrich Co. Preparation of thymol oil microemulsion (10% oil in water) was done in nanomaterial research and synthesis unit by using the method according to [26].

*(2) Characterization of Thymol Microemulsion*. Characterization of thymol microemulsion was done using Zetasizer Malvern Instrument (Corp, Malvern, UK) used to measure droplet size, surface charge (zeta potential), size distribution (polydispersity indexes, PDI), and electrical conductivity of the microemulsion. High-resolution transmission electron microscopy (HRTEM) observations were performed with a JEM 1400F HRTEM at a beam energy of 300 keV. Thymus oil and microemulsion components were analyzed using GC-MS at Central Laboratory in Faculty of Agriculture, Cairo University.

*(3) Cytotoxicity Assay*. Cell viability was assessed by SRB assay with different concentrations (0.01, 0.1, 1, 10, and 100 ug/ml) according to [27] using Vero cell (Green monkey cell line) obtained from Nawah Scientific Inc. (Mokatam, Cairo, Egypt). Cells were maintained in DMEM media supplemented with 100 mg/mL of streptomycin, 100 units/mL of penicillin, and 10% of heat-inactivated fetal bovine serum in humidified 5% (v/v) CO_2_ atmosphere at 37°C.

#### 2.1.3. Drug Used in In Vivo Experiments

Cotrimazine® was obtained from ADWIA Company, Egypt. It is administered at a dose of 33.34 mg of SDZ and 6.67 mg of TMP/kg b.wt orally for 5 days [28].

### 2.2. Experimental Model Design

#### 2.2.1. Birds Used in In Vivo Challenge

One-day-old Cobb broiler chickens were used in this trial. Chicks were housed in semiclosed house batteries. The chickens were provided with 24 hours' light throughout the first three days, then 23 hours' light, and 1 hour' dark until slaughter time (35 days). Chickens were fed a commercial starter diet (23% crude protein and 3000 kcal ME/kg diet) during the first two weeks of age and then a commercial grower diet (22% crude protein and 3150 kcal ME/kg diet). No antibiotics were added to the ration. No vaccination program was supported to the trial. Feed and water were available ad libitum.

*(1) Salmonella Enteritidis Strains Used in In Vivo Challenge*. Bacteriologically, serologically, and molecularly identified avian strain of *Salmonella* Enteritidis was obtained from Reference Laboratory for Veterinary Quality Control of Poultry Production (RLQP), Dokki, Egypt.

The first strain was sensitive to sulpha-trimethoprim, while the second one was multidrug resistant (MDR) strain especially to sulpha-trimethoprim. Stock cultures on tryptic soya agar (TSA) were stored at 4°C. Three milliliters of TSB was added to a sterile test tube. A loop of bacteria culture from a TSA plate was added to the media. The test tube was then capped and placed in a 37°C incubator for 18 to 24 h.

Colonies of *Salmonella* grew to approximately 1 × 10^9^ cfu/mL on average and were diluted according to McFarland standard in normal saline to inoculate 1 ml/chick once orally [29] at 15-day-old chicks.

*(2) Antimicrobial Activity of Essential Oil and the Nanoproduct (In Vitro Challenge)*. The disk diffusion method was applied for the determination of the antibacterial activity of the thyme oil as an essential oil and thyme microemulsion product according to [30]. The result measured according to the bacterial growth inhibition was the diameter of the zones (mm) without bacterial growth. For the interpretation of the results, four groups of inhibitory zones were examined: (i) no inhibition, (ii) <12 mm (weak activity zone), (iii) 12 mm ≥ inhibition zone <20 mm (intermediate activity), and (iv) inhibition zone ≥20 mm (strong activity).

#### 2.2.2. Experimental Design

Two hundred and fifteen one-day-old Cobb broiler chicks were kept for 49 days. At arrival we examined the paper lining of all chicks' boxes and organs (liver, yolk, and cecum) separately of five sacrificed chicks which are humanly euthanized and cultured to confirm their freedom from *Salmonella* strains. Chicks were randomly divided into fourteen groups (1 to 14). Each group consists of fifteen chicks as shown in (Table 1).

Chicks in groups one to twelve were orally inoculated with single dose of 1 × 10^9^ cfu/1 ml of *Salmonella* Enteritidis strains on day fifteen. On the eighteen day, the sulpha-trimethoprim was orally administered in a dose of 10 mg/kg b.wt/5 days while thymol oil and microemulsion were given in the drinking water in a dose of 1 ml/1 liter water/5 days after inoculation of both *Salmonella* Enteritidis strains by three days. This challenge was done in period from December 2020 to January 2021.

#### 2.2.3. Laboratory Examination

*(1) Enumeration of Salmonella Enteritidis*. Five cecal contents and fecal dropping from each group at 35, 42, and 49 days of age were examined for *Salmonella* Enteritidis enumeration [31]. Decimal dilutions in BPW were prepared and 0.1 mL of each dilution was inoculated by spread plate to XLD in duplicate. These plates were incubated for 24 h at 37°C and pink colonies with dark center were counted as *Salmonella*.

*(2) Sample Extraction for Detection of the Antibiotic Residue*. The extraction was done according to [32]. Tissues were homogenized and accurately weighed (3 g of muscle, 0.5 g of liver, and 0.25 g of kidney) in a 50 mL plastic centrifuge tube. Thirty mL of dichloromethane were added and the samples were homogenized with an Ultra Turrax for 1 min and centrifuged at 3000 g for 10 min. Supernatant was filtered through filter paper and collected. Ten mL of this liquid was transferred to a 15 mL glass centrifuge tube; 1 mL of hydrochloric acid 3 N was added, vortexed for 15 sec, and centrifuged for 5 min at 3000 g. From the upper aqueous layer, 250 *μ*L was transferred to a 15 mL glass centrifuge tube and another 250 *μ*L of 3.8 M sodium acetate solution was added and vortexed for 15 sec. Finally, a 100 *μ*L aliquot was injected into the LC system.

*(3) Reagents, Equipment and Chromatographic Conditions for Detection of the Antibiotic Residues*. All chemicals and reagents were of analytical grade. Analytical standards of SDZ and TMP were from Sigma Co. Liquid Chromatography (LC) Agilent Series 1200 was used. The chromatographic column was a reversed phase Venusil XBP C8 column (Agela Technologies, 4.6 mm i.d., 250 mm, 5 *μ*m) (Table 2).

*(4) Histopathological Examination*. Tissue specimens (heart, liver, spleen, proventriculus, and cecum) were collected from three chickens at second weak after treatment with antibiotics, thyme oil, and microemulsion. All organs were fixed in 10% neutral buffered formalin and processed by paraffin embedding technique for histopathological examination according to [33].

#### 2.2.4. Statistical Analysis

Statistical analysis was done using SPSS IBM 21 software and data were analyzed using statistical analysis system software (One-Way ANOVA). Results of all tests used in this study were considered significant at *P* value ≤ 0.05.

## 3. Results

### 3.1. Characterization of Thymol Microemulsion

**Figure 1:**
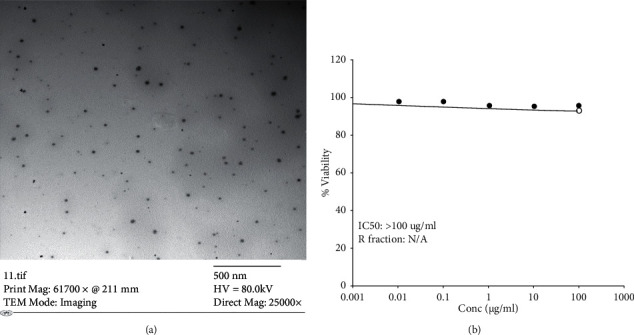
(a) TEM of thymol microemulsion. (b): Cell viability % of thymol microemulsion effect on Vero cells.

Microemulsion characterization of the nanodroplet was mainly determined by TEM whose size is 28.65 ± 0.89 nm with a narrow size distribution (polydispersity index: 0.28) which indicated that greater homogeneity can be realized (Figure 1(a)).

The zeta potential is an indicator that stable suspensions are generally taken by using dynamic light scattering (DLS) of a −2.6 ± 8.32 mV and conductivity 0.089 ms/cm.

When GC-Mass analyzed the thymol oil it had 9 components which were o-Cymene (4.98%), Geraniol (1.55%), Camphor (1.35%), Thymol (24.68%), Hexadecanoic acid (24.24%), vaccenic acid (11.54%), cis-vaccenic acid (26.07%), C10H9Cl9 (1.62%), and 1,3-Diolein (1.26%), while thymus oil microemulsion has 14 components which are Citronellol (4.19%), Menthol(3,10%), *α*–Guaiene (2.60%), Eupatorin (15.80%), Tribehenin (13.56%), 1,2-Dipalmitoyl-rac-glycerol (8.51%), Ayanin (8.62%), 2-Ethyldecyl 2-ethylundecyl phthalate (11.28%), *α*-Sitosterol (1.80%), *β*-Sitosterol (10.5%), Anthracene (10.98%), Tridecanoic acid (2.97%), Corynoxine (2.04%), and Marinosin (2.33%).

On the confluent surface of Vero cells, results for thymol microemulsion had different concentration (0.01, 0.1, 1, 10, and 100 ug/ml) after 3 days of inoculation; the effect on cell viability% was assessed by SRB assay to be 98.48%, 98.21%, 96.42%, 95.80%, and 95.73%, respectively, in 100 ug/ml and IC50 > 100 ug/ml (Figure 1(b)).

### 3.2. In Vitro: Antibacterial Activity of Thymol Oil and Microemulsion

Antimicrobial activity of thymol oil and microemulsion to *Salmonella* Enteritidis strain was done by measuring the diameter of inhibitory zone of bacterial growth surrounding the disc of three tested concentrations (0.5, 1.0, and 5.0 ml) according to [29] which was 15, 25, and 50 millimeters, respectively, for thymol oil while microemulsion results were 17, 32, and 60 ml. In the in vivo challenge, we choose the concentration of 1 ml/liter which represented thymol oil dose (0.1%) and microemulsion dose (0.01%).

### 3.3. Experimental Results

#### 3.3.1. Mortality Rate Results

High mortality rates were detected in group one (which was only inoculated with *Salmonella* Enteritidis MDR strain), group two (inoculated with *Salmonella* Enteritidis strain sensitive to sulpha-trimethoprim group), and group three (inoculated with MDR *Salmonella* Enteritidis strain and treated with sulpha-trimethoprim antibiotics) to be 40%, 26.7%, and 20%, respectively; meanwhile, the other groups show a significant decrease in the mortality rate reaching 6.7% after treatment with thymol oil dose (0.1%) and microemulsion dose (0.01%) and sulpha-trimethoprim, as shown in Table 3.

#### 3.3.2. Results of *Salmonella* Enteritidis Count during Experiment

Results of the fourteen groups showed high count of *Salmonella* Enteritidis in positive groups. Sulpha-trimethoprim antibiotic has great effect on group inoculated with *Salmonella* sensitive strain while it has no effect on the group which was inoculated with *Salmonella* Enteritidis MDR strain. Other groups which were treated with thymol oil and thymol microemulsion with or without antibiotics showed lower count as shown in Table 4.

*(1) Statistical Analysis of Total Salmonella Enteritidis Count in Cecal Content*. The statistical analysis was done for total *Salmonella* Enteritidis count of cecal content of three chickens, from each group which were infected with *Salmonella* MDR strain, and other groups which were infected with *Salmonella* Enteritidis sensitive to sulpha-trimethoprim (sxt). The results of the count of *Salmonella* Enteritidis sensitive strain from both the second and fourth weeks showed a highly significant difference between groups which marked by (^∗^) at the same column at *P* value ≤ 0.05, as shown in Tables 5 and 6.

#### 3.3.3. Measuring Sulfadiazine and Trimethoprim Residuals in Different Boiler Tissues

The distributions of sulfadiazine and trimethoprim in different boiler tissues after administration of 33.34 and 6.67 mg/kg b.wt for 5 days, respectively, are illustrated in Table 7 for sulfadiazine and in Table 8 for trimethoprim. The results showed that both compounds were rapidly distributed in all examined tissues.

#### 3.3.4. Histopathological Results

**Figure 2:**
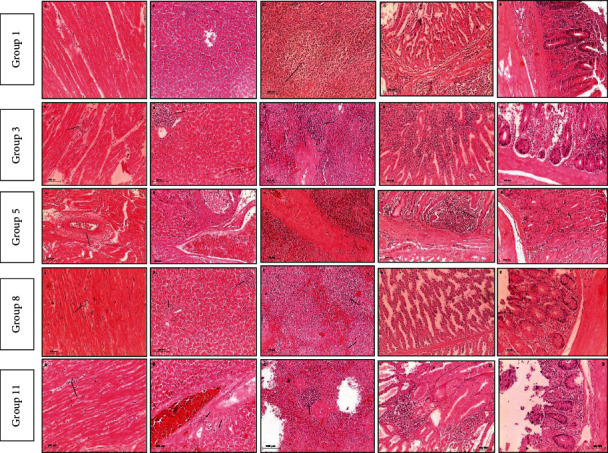
Microscopic picture of infected groups with MDR *Salmonella* Enteritidis strain.

**Figure 3:**
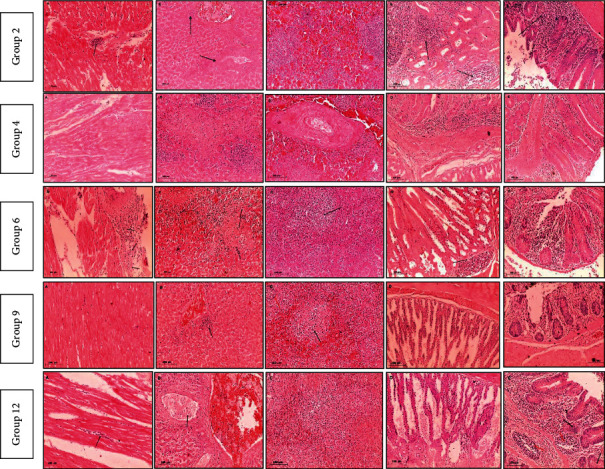
Microscopic picture of infected groups with SXT *Salmonella* Enteritidis sensitive strain.

Microscopic examination of negative control as well as treated groups with microemulsion dose (0.01%) and thyme oil dose (0.1%) revealed normal histological architecture of examined organs. Meanwhile, variable grades of pathological alterations were recorded in examined organs from infected/and or treated groups described in Tables 9 and 10 and Figures 2 and 3.

In case of treated groups that were infected with resistant strain (3, 5, 8, and 11), group 8 showed less pathological alterations than other treated challenged groups even in case of antibiotic treated group. On the other hand, equivalent finding was recorded in group 9 which was infected with sensitive strain. That finding means remarkable histopathological improvement in case of treatment with 0.1% thyme oil dose which alleviates inflammatory reaction and tissue destruction induced by *Salmonella* Enteritidis even in case of antibiotic treatment which has a hazard effect on the tissue.

Figure 2 shows pathological changes in infected groups with MDR 2 weeks PI (A: heart, B: liver, C: spleen, D: proventriculus, and E: cecum).

Group 1 shows (A) mild myocardial edema, (B) focal lymphocytic cells infiltration and congested sinusoids, (C) lymphocytic depletion (arrow), (D) normal histological architecture, and (E) typhlitis with marked lymphocytic cells infiltration. Group 3 shows (A) myocarditis with marked lymphocytic cells infiltration in between myofibril (arrow), (B) degenerative hepatocytes with focal aggregation of lymphocytic cells (arrow), (C) splenitis with 2ry lymphocytic follicle formation (arrow) and vasculitis (red arrow), (D) severe inflammatory cells infiltration with necrotic mucosa, and (E) severe typhlitis with marked destruction of the mucosa replaced with inflammatory cells. Group 5 shows (A) severe myocarditis with vasculitis (arrow) and diffuse inflammatory cells infiltration, (B) hepatic cells degeneration with necrosis of blood vessel wall, (C) marked splenitis with severe vasculitis, (D) marked proventriculitis with severe destruction of mucosa replaced with inflammatory cells (arrow), and (E) mild typhlitis with hyperplasia of cecal glands (arrow). Group 8 shows (A) mild myocarditis with edema and focal inflammatory cells infiltration (arrow), (B) mild hepatocytic cells degeneration with focal inflammatory cells aggregation (arrow), (C) mild lymphocytic cells depletion with histiocytic cells infiltration (arrow), (D) normal picture of proventriculus, and (E) mild inflammatory reaction. Group 11 shows (A) mild myocarditis with inflammatory cells infiltration (arrow), (B) moderate hepatitis with lymphocytic and histiocytic cells infiltration (arrow) and necrotic areas and congested blood vessels, (C) severe splenitis with multiple vasculitis (red arrow) and 2ry lymphocytic cells follicle formation (arrow), (D) severe proventriculitis with destructed mucosa replaced with inflammatory cells, and (E) typhlitis with massive inflammatory cells infiltration and necrosis.

Figure 3 shows pathological changes in infected groups with SXT 2 weeks PI (A: heart, B: liver, C: spleen, D: proventriculus, and E: cecum).

Group 2 shows (A) myocardial necrosis with lymphocytic cells infiltration (arrow), (B) necrosis of vascular wall with lymphocytic cells infiltration (arrow), (C) marked lymphocytic depletion, (D) proventriculitis with massive lymphocytic cells infiltration (arrow), and (E) severe typhlitis with marked lymphocytic cells infiltration (arrow). Group 4 shows (A) mild myocarditis with interstitial edema mixed with inflammatory cells, (B) large necrotic area with multiple lymphocytic cells infiltration, (C) severe splenitis with marked vasculitis and hemorrhages, (D) destruction of mucosal surface replaced by inflammatory cells infiltration, and (E) massive inflammatory cells infiltration causing atrophy of cecal gland. Group 6 shows (A) marked pericarditis (arrow) and necrosis of myofibrils, (B) marked necrosis of hepatic parenchyma, (C) mild lymphocytic depletion (arrow), (D) proventriculitis with inflammatory cells infiltration (arrow) and degeneration of lining epithelium (red arrow), and (E) moderate typhlitis with atrophy of cecal glands. Group 9 shows (A) normal architecture of myocardium, (B) congestion of hepatic blood vessels with perivascular aggregation of inflammatory cells (arrow), (C) mild lymphocytic cells depletion with heterophilic cells infiltration (arrow) and 2ry lymphocytic follicle (red arrow), (D) normal picture of proventriculus, and (E) typhlitis with atrophied cecal gland. Group 12 shows (A) mild myocarditis with inflammatory cells infiltration in between myofibrils (arrow), (B) marked congested blood vessels (red arrow) with infiltration of lymphocytes and heterophils inside blood vessel (arrow), (C) mild lymphocytic depletion, (D) mild proventriculitis with desquamation of epithelial cells mixed with inflammatory cells and debris, and (E) typhlitis with atrophied cecal gland (arrow) and lymphocytic depletion (red arrow).

## 4. Discussion

*Salmonella* infection is one of the major bacterial diseases in poultry, which economically has an adverse effect on cost of the treatment especially in case of multidrug resistance strains [34]. This study aimed to investigate, in vitro and in vivo, the effect of antibiotics compared with thymol and microemulsion on antibiotic-resistant bacteria. Thymol was found to have a positive effect on significantly reducing the number of antibiotic-resistant bacteria, which may make it a good alternative to antibiotics. This natural product could be an efficient alternative that would have positive effect to fight antimicrobial resistance that is observed in human which originates from the administering of antibiotics to farm animals [10, 35, 36].

The fabrication of microemulsion with lesser droplet size in the presence of double bonds in the nonpolar chain of nonionic surfactants obtained results in agreement with different results [37]. The conductivity of the microemulsions increased by increasing essential oil concentration which demonstrated that water is the continuous phase (*P* < 0.05). This is due to the fact that conductivity of the solutions is directly proportional to the amount of ions and increases by increasing the ions. The cell viability reached 88–90% for microemulsions containing thyme and rosemary oil, respectively, which showed that prepared microemulsions are safe and nontoxic [38].

Kumari et al. [39] reported that in the stability study for the characterization of thymol based microemulsions they had spherical droplets size (293 ± 2.7 nm), PDI (0.15), and zeta potential (−32 mV) in 50 min sonicated as compared to other microemulsions. The results are strengthened by the fact that greater sonic energy flows to emulation through greater surfactant adsorption on hydrophobic droplet surfaces and helps to reduce the droplet dimensions and the spread of small droplet. Diffusion or movement of solubilized oil molecules from small droplets to large droplets through dispersed droplets causes dispersion of droplets to grow with reduced size (Ostwald ripening method).

The optimized 0.82% of thymus oil microemulsions was pale yellow to amber transparent microemulsion with a globule size of 14.23 ± 0.3 nm, zeta potential of −0.69 mV, and PDI value of 0.00143 indicating a stable microemulsion [40].

### 4.1. In Vitro Experiment

In our study, disc diffusion method is used for determination of doses and concentration of thyme oil and nanomaterial emulsion which affected the *Salmonella* strains used in our experimental design which is the same as [30, 41], while minimum inhibitory concentration method (MIC) is used in the detection of the effective concentration which affects *Salmonella* strain in this study.

“In in vivo challenge in this study, the mortality rates were 40% in the positive control while other groups which were treated with the antibiotic and thymol oil and the microemulsion show lower percentage of 6.7% which is in accordance with [43] who reported that, after challenge with *Salmonella* and treatment with thymol oil and its microemulsion, the mortality rate among chicken treated with thymol oil and its microemulsion was 3%.”

Paper [44] did not find any significant difference in mortality rates of groups which were treated with essential oil and positive control.

In the present study, there was a little *Salmonella* count in cecal content when adding thymol oil in the water of chickens; these results agreed with [19, 44]. On the other hand, [42] did not find any significant decrease in the *Salmonella* count in cecal content when treated with thymol oil. In our study, we found the effect of thymol microemulsion was higher than thymol oil on the percentage of reduction in the count of *Salmonella* in cecal content, which is similar to [25] who found better effect of 0.5% concentration of thymol microemulsion than 1% of thymol oil on reduction of *Salmonella* count in cecal content.

As a result of threats of drug residues in foods industry of animal origin, limits for these residues have been set by the competent authorities and bodies as the European Union has set MRL of residues of both SDZ and TMP in different poultry tissues, 100 and 50 ppb, respectively [45, 46]. After administration of SDZ and TMP at a doses of 33.34 and 6.67 mg/kg b.wt, respectively, for 5 days, SDZ residues were still detected till the 2nd day in muscle and till the 5th day in liver and kidneys, but TMP residues were still detected till the 2nd day in muscle, the 4th day in liver, and the 5th day in kidneys after cessation of treatment in healthy broiler chickens; these findings agree with that detected in broilers by [47], but are not in the same line with that noted by [48] who revealed that TMP residues were still detected only till the 2nd day in muscle, liver, and kidney in healthy broiler chickens and this may rely on using different dose at 33.4 g/l for just 3 consecutive days. Infection with MDR and SS strains in the other four groups significantly decreases the SDZ concentrations in the muscle at the 2nd day and in liver and kidneys at 4th day and significantly decreases the TMP concentrations in analyzed tissues to be detected in the muscle at the 2nd day and in liver and kidneys at 3rd day after last oral dose. The obtained data revealed that the recommended withdrawal time of SDZ and TMP in healthy broilers according to the accepted guidelines is 5 days and this agrees with [49] who found that the withdrawal period for such an oral formulation of SDZ and TMPt in healthy pigs should not be less than 5 days using different animal species and this also agrees with [48] who revealed that residue concentrations of SQX and TMP were lower than their maximum residual limits (MRLs) in all tissues at 5 days after the treatment.

In our study we found no histopathological effects of thymol oil and thymol microemulsion in noninfected groups while the infected groups which were treated with thyme oil show lower histopathological changes than those treated with thymol microemulsion and antibiotic and these results were similar to [35]. On the other hand our results disagree with what is mentioned by [25] who found better histopathological improvement in cecal wall of infected chicken with *Salmonella* after treatment with the thymol microemulsion than after treatment with thymol oil.

## 5. Conclusion

Thymol oil and thymol microemulsion have antibacterial effect, which makes reduction in *Salmonella* count in broiler cecum after administration of thymol and its microemulsion as a treatment to the infected chicken with *Salmonella* Enteritidis even with the multidrug resistance strain. Treatment with thymol oil makes better improvement in histopathological picture of infected chicken than the thymol microemulsion. By robust HPLC method, the SDZ and TMP residues in tissues of infected groups treated with Cotrimazine® + thyme oil microemulsion had slight significant economic impact (*P* < 0.05) compared to Cotrimazine® alone.

## 6. Recommendations

A further study with increasing the thymol oil and thymol microemulsion concentration dose and/or increasing treatment period is recommended. The recommended withdrawal time of SDZ and TMP in healthy broilers is 5 days and in infected broilers with MDR and SS strains alone and in presence of thyme oil is four days to be safe for human consumption.

## Figures and Tables

**Table 1:** Identification of groups of chicken in the in vivo challenge.

GP1	Inoculated with *Salmonella* Enteritidis multidrug resistance strain (MDR) Strain 1
GP2	Inoculated with *Salmonella* Enteritidis sensitive to sulpha-trimethoprim strain (SXT) Strain 2
GP3	Strain 1 + sulpha-trimethoprim antibiotic
GP4	Strain 2 + sulpha-trimethoprim antibiotic
GP5	Strain 1 + sulpha-trimethoprim antibiotic + microemulsion
GP6	Strain 2 + sulpha-trimethoprim antibiotic + microemulsion
GP7	Only thymol oil
GP8	Strain 1 + thymol oil
GP9	Strain 2 + thymol oil
GP10	Only microemulsion
GP11	Strain 1 + microemulsion
GP12	Strain 2 + microemulsion
GP13	Negative control
GP14	Only antibiotic

**Table 2:** Reagents, equipment, and chromatographic conditions for detection of the antibiotic residues.

Injection volume	100 *μ*L
Flow rate	1 mL/min
Mobile phase	Acetonitrile: 10 mM phosphoric acid (16:84 v/v)
Column temp	Ambient temperature
UV	271 nm
Injection volume	100 *μ*L

**Table 3:** Total number and percentage of mortality rates in each group.

Groups	Number of dead chicken in each group^*∗*^(*n* = 15)	Percentage of mortality rates
Group 1	6	40
Group 2	4	26.70
Group 3	3	20
Group 4	1	6.70
Group 5	1	6.70
Group 6	1	6.70
Group 7	1	6.70
Group 8	1	6.70
Group 9	1	6.70
Group 10	1	6.70
Group 11	1	6.70
Group 12	1	6.70
Group 13	1	6.70
Group 14	1	6.70

^*∗*^(*n* = 15) total mean of chicken in each group.

**Table 4:** Results of effect of total *Salmonella* Enteritidis count in in vivo challenge.

Groups	Count of *Salmonella* Enteritidis from cecum		Count of *Salmonella* Enteritidis from fecal dropping	
	2nd week PI (postinoculation) (age of chicken 35 days)	4th week PI (postinoculation) (age of chicken 49 days)	3rd week PI (postinoculation) (age of chicken 42 days)	4th week PI (postinoculation) (age of chicken 49 days)
GP1	Over 300 × 10^2^	Over 300 × 10^2^	Over 300 × 10^2^	Over 300 × 10^2^
GP2	Over 300 × 10^2^	Over 300 × 10^2^	Over 300 × 10^2^	Over 300 × 10^2^
GP3	Over 300 × 10^2^	Over 300 × 10^2^	Over 300 × 10^2^	Over 300 × 10^2^
GP4	0	0	0	0
GP5	10 × 10^2^	Less than 1 × 10^1^	19 × 10^2^	5 × 10^2^
GP6	0	0	0	0
GP8	Over 300 × 10^2^	18 × 10^2^	Over 300 × 10^2^	20 × 10^2^
GP9	Over 300 × 10^2^	10 × 10^2^	Over 300 × 10^2^	25 × 10^2^
GP11	12 × 10^2^	8 × 10^2^	Over 300 × 10^2^	15 × 10^2^
GP12	0	0	8× 10^2^	0

**Table 5:** Statistical analysis of total *Salmonella* Enteritidis count in groups challenged with MDR (log of mean ± SD).

Group	2nd week PI	4th week PI
1	4.48 ± 0^*∗*^	4.48 ± 0^*∗*^
3	4.48 ± 0	4.48 ± 0
5	1.67 ± 1.52	0^*∗*^
8	4.48 ± 0	2.03 ± 1.77^*∗*^
11	1.73 ± 1.53^*∗*^	1.03 ± 1.77^*∗*^

There is a highly significant difference between groups having ^*∗*^at the same column at *P* value ≤ 0.05.

**Table 6:** Statistical analysis of total *Salmonella* Enteritidis total count in groups challenged with SXT (log of mean ± SD).

Group	2nd week PI	4th week PI
2	4.48 ± 0^*∗*^	4.48 ± 0^*∗*^
4	0^*∗*^	0^*∗*^
6	0^*∗*^	0^*∗*^
9	4.22 ± 0.44	1 ± 1.73^*∗*^
12	0^*∗*^	0^*∗*^

There is a highly significant difference between groups having ^*∗*^at the same column at *P* value ≤ 0.05.

**Table 7:** Results of sulfadiazine concentrations (*μ*g/gm) in different boiler tissues after administration of 33.34 mg/kg b.wt. for 5 days.

Tissue	Groups	1st day	2nd day	3rd day	4th day	5th day	MRL
Muscle	Antibiotic in normal healthy group	110 ± 9	47.3 ± 3.1	ND	ND	ND	100
	Antibiotic + MDR infected group	86 ± 6^*∗*^	31 ± 3^*∗*^	ND	ND	ND	
	Antibiotic + SS infected group	88 ± 6^*∗*^	33 ± 3^*∗*^	ND	ND	ND	
	Antibiotic + MDR infected group fed on thyme microemulsion	84 ± 5^*∗*^	29 ± 2^*∗*^	ND	ND	ND	
	Antibiotic + SS infected group fed on thyme microemulsion	83 ± 6^*∗*^	27 ± 2^*∗*^	ND	ND	ND	
Liver	Antibiotic in normal healthy group	196 ± 8	119 ± 10	86 ± 7	63 ± 10	36 ± 5	
	Antibiotic + MDR infected group	142 ± 11^*∗*^	99 ± 7^*∗*^	72 ± 4^*∗*^	45 ± 4^*∗*^	ND	
	Antibiotic + SS infected group	144 ± 10^*∗*^	100 ± 8^*∗*^	72 ± 3^*∗*^	47 ± 3^*∗*^	ND	
	Antibiotic + MDR infected group fed on thyme microemulsion	139 ± 11^*∗*^	97 ± 6^*∗*^	67 ± 2^*∗*^	43 ± 4^*∗*^	ND	
	Antibiotic + SS infected group fed on thyme oil microemulsion	134 ± 9^*∗*^	94 ± 6^*∗*^	66 ± 3^*∗*^	40 ± 2^*∗*^	ND	
Kidney	Antibiotic in normal healthy group	515 ± 53	342 ± 26	294 ± 13	128 ± 11	66 ± 6	
	Antibiotic + MDR infected group	391 ± 10^*∗*^	195 ± 14^*∗*^	110 ± 11^*∗*^	78 ± 4^*∗*^	ND	
	Antibiotic + SS infected group	395 ± 16^*∗*^	193 ± 14^*∗*^	112 ± 6^*∗*^	81 ± 6^*∗*^	ND	
	Antibiotic + MDR infected group fed on thyme microemulsion	387 ± 13^*∗*^	188 ± 6^*∗*^	104 ± 12^*∗*^	74 ± 5^*∗*^	ND	
	Antibiotic + SS infected group fed on thyme microemulsion	386 ± 19^*∗*^	187 ± 12^*∗*^	103 ± 10^*∗*^	75 ± 4^*∗*^	ND	

ND: not detected. ^*∗*^Significant with normal healthy group given antibiotic only at the same column using ANOVA at *P* ≤ 0.05.

**Table 8:** Results of trimethoprim concentrations (*μ*g/gm) in different boiler tissues after administration of 6.67 mg/kg b.wt. for 5 days.

Tissue	Groups	1st day	2nd day	3rd day	4th day	5th day	MRL
Muscle	Antibiotic in normal healthy group	41 ± 4	18 ± 1	ND	ND	ND	50
	Antibiotic + MDR infected group	26 ± 2^*∗*^	ND	ND	ND	ND	
	Antibiotic + SS infected group	26 ± 1^*∗*^	ND	ND	ND	ND	
	Antibiotic + MDR infected group fed on thyme microemulsion	26 ± 3^*∗*^	ND	ND	ND	ND	
	Antibiotic + SS infected group fed on thyme microemulsion	25 ± 1^*∗*^	ND	ND	ND	ND	
Liver	Antibiotic in normal healthy group	68.6 ± 3	42 ± 4	30 ± 3	22 ± 4	ND	
	Antibiotic + MDR infected group	50 ± 5^*∗*^	28 ± 1^*∗*^	ND	ND	ND	
	Antibiotic + SS infected group	52 ± 2^*∗*^	28 ± 1^*∗*^	ND	ND	ND	
	Antibiotic + MDR infected group fed on thyme microemulsion	50 ± 7^*∗*^	27 ± 3^*∗*^	ND	ND	ND	
	Antibiotic + SS infected group fed on thyme microemulsion	51 ± 4^*∗*^	27 ± 3^*∗*^	ND	ND	ND	
Kidney	Antibiotic in normal healthy group	346 ± 35	230 ± 19	195 ± 11	86 ± 6	44 ± 4	
	Antibiotic + MDR infected group	219 ± 9^*∗*^	134 ± 9^*∗*^	87 ± 3^*∗*^	ND	ND	
	Antibiotic + SS infected group	219 ± 9^*∗*^	132 ± 10^*∗*^	88 ± 4^*∗*^	ND	ND	
	Antibiotic + MDR infected group fed on thyme microemulsion	217 ± 6^*∗*^	134 ± 10^*∗*^	88 ± 3^*∗*^	ND	ND	
	Antibiotic + SS infected group fed on thyme microemulsion	380 ± 19^*∗*^	187 ± 12^*∗*^	90 ± 3^*∗*^	ND	ND	

ND: not detected. ^*∗*^Significant with normal healthy group given antibiotic only at the same column using ANOVA at *P* ≤ 0.05.

**Table 9:** Pathological lesions in groups infected with MDR strain 2 weeks after inoculation.

Organ	Lesion	Score				
		GR1	GR3	GR5	GR8	GR11
Heart	Edema	+++	+++	+++	+	+++
	Hemorrhage	+++	++	+++	—	+++
	Necrosis	++	++	+	—	+
	Heterophilic infiltration	++	+++	++	+	++
	Pericarditis	+	+	—	—	—

Liver	Heterophilic infiltration	++	+++	++	+	++
	Congestion of blood vessels and sinusoids	++	++	+++	+	+++
	Hepatocytic degeneration	++	++	++	+	++
	Necrosis	+	++	—	—	—

Spleen	Lymphocytic depletion	+	+	++	+	++
	Vasculitis	+	++	+++	+	+++
	2ry follicle	+	+	—	—	+
	Necrosis	—	+	—	—	—

Proventriculus	Heterophilic infiltration	+++	+++	+++	++	+++
	Mucosal degeneration	+++	+++	++	+	++
	Necrosis	++	++	++	—	++

Cecum	Thickening wall	+++	++	++	+	++
	Heterophilic infiltration	+++	+++	+++	++	+++
	Necrosis	++	—	+	—	+

(+ = mild, ++ = moderate, +++ = severe).

**Table 10:** Pathological lesions in groups treated with SXT strain 2 weeks after inoculation.

Organ	Lesion	Score				
		GR2	GR4	GR6	GR9	GR12
Heart	Edema	+++	++	++	+	++
	Hemorrhage	+++	++	++	+	++
	Necrosis	++	—	—	—	++
	Heterophilic infiltration	++	+	+	+	++
	Pericarditis	+	—	++	—	—

Liver	Heterophilic infiltration	++	+++	+++	++	+++
	Congestion of blood vessels and sinusoids	+++	+++	+++	++	+++
	Hepatocytic degeneration	++	+++	+++	++	+++
	Necrosis	+	++	++	+	+++

Spleen	Lymphocytic depletion	++	+++	++	+	+
	Vasculitis	++	+++	+++	+	+
	2ry follicle	+	—	+++	+	+
	Necrosis	++	+++	—	—	—

Proventriculus	Heterophilic infiltration	+++	+++	+++	+	++
	Mucosal degeneration	+++	+++	+++	+	+++
	Necrosis	++	++	+++	—	+

Cecum	Thickening wall	+++	+++	+++	++	+++
	Heterophilic infiltration	+++	+++	+++	++	+++
	Necrosis	++	++	+++	++	+++

(+ = mild, ++ = moderate, +++ = severe).

## Data Availability

All data are included within the manuscript.
